# Scale-dependent effects of habitat fragmentation on the genetic diversity of *Actinidia chinensis* populations in China

**DOI:** 10.1038/s41438-020-00401-1

**Published:** 2020-10-13

**Authors:** Wenhao Yu, Baofeng Wu, Xinyu Wang, Zhi Yao, Yonghua Li, Yongbo Liu

**Affiliations:** State Key Laboratory of Environmental Criteria and Risk Assessment, Chinese Research Academy of Environmental Sciences, 8 Dayangfang, 100012 Beijing, China

**Keywords:** Molecular ecology, Genetic markers, Population genetics

## Abstract

Spatial scale partly explains the differentiated effects of habitat fragmentation on plant biodiversity, but the mechanisms remain unclear. To investigate the effects of habitat fragmentation on genetic diversity at different scales, we sampled *Actinidia chinensis* Planch. at broad and fine scales, China. The broad-scale sampling included five mountain populations and one oceanic island population (Zhoushan Archipelago), and the fine-scale sampling covered 11 lake islands and three neighboring land populations in Thousand-Island Lake (TIL). These populations were genotyped at 30 microsatellite loci, and genetic diversity, gene flow, and genetic differentiation were evaluated. Genetic differentiation was positively related to geographical distance at the broad scale, indicating an isolation-by-distance effect of habitat fragmentation on genetic diversity. The oceanic population differed from the mainland populations and experienced recent bottleneck events, but it showed high gene flow with low genetic differentiation from a mountain population connected by the Yangtze River. At the fine scale, no negative genetic effects of habitat fragmentation were found because seed dispersal with water facilitates gene flow between islands. The population size of *A. chinensis* was positively correlated with the area of TIL islands, supporting island biogeography theory, but no correlation was found between genetic diversity and island area. Our results highlight the scale-dependent effects of habitat fragmentation on genetic diversity and the importance of connectivity between island-like isolated habitats at both the broad and fine scales.

## Introduction

Habitat fragmentation caused by anthropogenic activities is considered a key factor threatening biodiversity^1,2^. Habitat fragmentation affects biodiversity by increasing isolation between populations and decreasing effective population size, which alters inbreeding and genetic drift within populations, as well as gene-flow frequency between populations^3–5^. This mechanism can be explained by island biogeography theory^6,7^.

Habitat fragmentation changes continuous habitats to isolated patches and results in the formation of island-like habitats. Guo defined island-like habitats as island entities or areas, such as mountains, oases, or discontinuous forests, isolated by farmlands^8^. Populations in island-like habitats are usually thought to have low genetic diversity^9,10^, which is explained by the species–area relationship from island biogeography theory^11^. Population size is positively related to island area, so small islands are likely influenced by genetic drift, which usually leads to low genetic diversity^12,13^. Habitat fragmentation can decrease gene flow among populations because low connectivity between habitats limits seed dispersal and insect pollination^14–16^. If gene flow is too low to buffer the negative effects of inbreeding and genetic drift in fragmented populations^17^, then habitat fragmentation may decrease population fitness and increase the risk of extinction^18,19^. Toczydlowski and Waller found high levels of inbreeding and genetic differentiation between isolated populations of *Impatiens capensis*^20^.

However, some studies did not find negative effects of habitat fragmentation on genetic diversity^21,22^. The differentiated effects of habitat fragmentation on genetic diversity may be associated with species characteristics (e.g., mating pattern), environmental features (e.g., landscape attributes)^23–25^, or the scale of habitat fragmentation^26,27^. Compared to monoecious plants, dioecious plants have higher potential for adaptation and higher genetic diversity because they have higher selection efficacy both against deleterious mutations and for beneficial mutations^28^. Long-lived species have higher potential against genetic erosion from habitat fragmentation than short-lived species because of the occurrence of gene flow among populations^29^. In a broad-scale study across Tibet, Sichuan, and Yunnan provinces in China (ca. 1 × 10^6^ km^2^), habitat fragmentation was found to weaken gene flow between populations and cause isolation-by-distance effects^30^, while there was a lack of evidence of isolation-by-distance effects in a fine-scale-fragmented habitat (ca. 1.5 km^2^) on Stephens Island, New Zealand^31^. Long-distance dispersal of seeds can promote gene flow and reduce the genetic distance between populations on a broad scale^32^. Thus, spatial scale and species characteristics, such as dioecy, a long lifespan, and long-distance dispersal ability, should be considered when studying the effects of habitat fragmentation on genetic diversity.

Here, we investigated the genetic diversity of *Actinidia chinensis* in China across its national distribution range, considering geographic distance at a broad scale (ca. 1 × 10^6^ km^2^) and a fine scale (ca. 30 km^2^). This study aimed to investigate (1) whether island-like isolated habitats limit gene flow and cause differentiation between populations, (2) whether the correlation between genetic diversity and island area in fragmented habitats supports island biogeography theory, and (3) whether the genetic effect of habitat fragmentation is scale-dependent. We employed microsatellites known as simple sequence repeats (SSRs) for genetic diversity analysis because they are codominant loci and have the advantages of a low cost, high polymorphism, high reliability, and a low requirement for DNA quality^33,34^, which facilitates the analysis of population demography in fine-scale regions^35^. These findings are expected to provide useful information for the conservation of genetic diversity.

## Materials and methods

### Plant materials and sampling regions

**Fig. 1:**
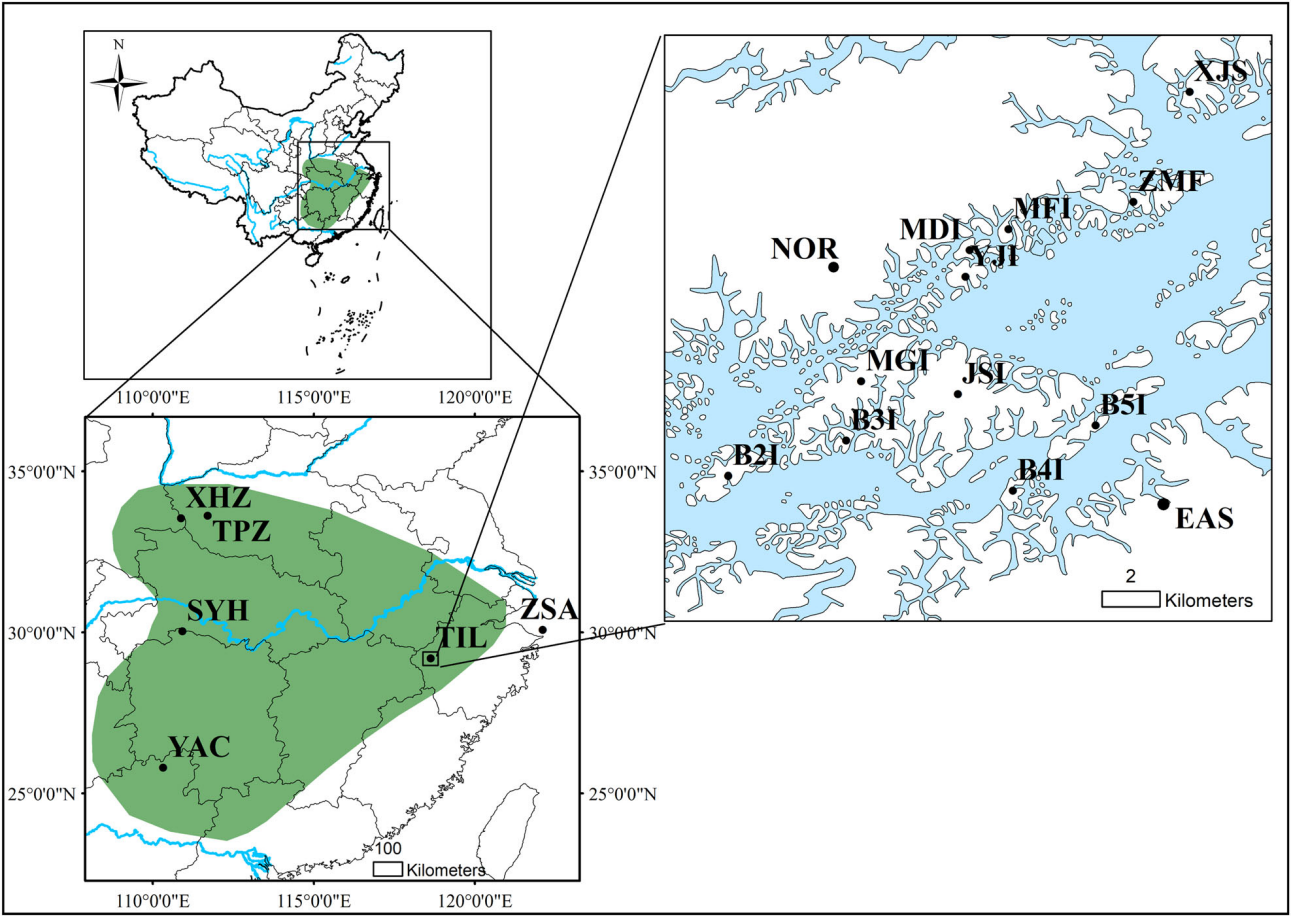
Distribution area and sampling sites of *A. chinensis* populations in China. The green shading in the left subfigures indicates the distribution range of wild *A. chinensis* populations in China. Lower-left subfigure, XHZ and TPZ (Henan Province in the north), SYH (Hunan Province in the west), and YAC (Guangxi Province in the south) were sampling points around the distribution edge of *A. chinensis* in China; TIL, Thousand-Island Lake in Zhejiang Province; ZSA, the Zhoushan Archipelago. Right subfigure, sampling points on 11 TIL islands and the neighboring mainland (the SOU site is not marked in the subfigure because it is located over 30 km away from the lake)

*A. chinensis* (2*n* = 2*X* = 58) is a perennial, dioecious, and deciduous liana^36^ with a wide distribution in China (Fig. 1). *A. chinensis* plants are cross-pollinated by insects or wind and disperse through seeds^37,38^. One-year-old shoots are gray-green brown and glabrous or sparsely covered with white downy hairs, while 2-year-old stems are dark brown and glabrous^39^. *A. chinensis* plants generally start to flower 3 years after seed germination.

Thousand-Island Lake (TIL, Fig. 1), located in eastern China (Chun’an County, Zhejiang Province), is an artificial lake constructed for impoundment water^40^. The islands of TIL were originally a range of continuous mountains near neighboring mainland and then became fragmented due to the construction of the Xin-An Jiang Reservoir in 1958^5^. The Zhoushan Archipelago (ZSA), located in the East China Sea, was originally a part of mainland China and formed by rising sea levels ca. 7000–9000 years ago^27^ (Fig. 1).

For the TIL fine-scale analysis, to test island biogeography theory, we investigated the abundance of *A. chinensis* on all TIL islands, except for the largest (JSI and MGI) islands, where population size was estimated by multiplying the average number of plants in three randomly sampled bays by the number of bays (Supplementary Table S1 and Fig. 1). At the same time, we measured the diameter at breast height (DBH) of *A. chinensis* plants. To infer plant age on the islands, we counted the tree rings of six *A. chinensis* plants and constructed a linear regression between tree rings and DBH. In addition, three neighboring land populations (north, east, and south) were sampled around the TIL islands (Fig. 1), which were used and labeled as one (TIL-M) population in the next analysis at a broad scale.

For the broad-scale analysis, we sampled *A. chinensis* on four mountains (TPZ, XHZ, YAC, and SYH) and one oceanic island (ZSA) in China (Fig. 1), with 15–40 plants per population. We sampled individuals at least 5 m from each other whenever possible.

A total of four to six fresh leaves of *A. chinensis* were sampled per plant. Half of the leaves per plant were dried by allochroic silica gel for DNA extraction, and the other half was kept fresh for flow cytometry.

### Ploidy detection

To determine the genetic background of *A. chinensis* plants, we detected the ploidy of samples with flow cytometry. Thirty milligrams of fresh leaves per plant were chopped and dissociated by 1 mL of HEPES buffer. After filtration and centrifugation, the dissociated products were dyed with 500 µl of PI (propidium iodide) solution for 10–15 min. Ploidy analysis was conducted by flow cytometry (BD LSRFortessa) with FSC 488, SSC 290, and PI 411–432 (adjusted based on the peak value of the control sample)^41^.

### DNA extraction and microsatellite genotyping

Whole-genomic DNA of *A. chinensis* was extracted from 25 mg of dry leaves using a Hi-DNA Secure Plant Kit (TIANGEN Biotech Co., Ltd.), using a modified version of the SDS method^5^. The genetic diversity of *A. chinensis* populations was detected using 30 microsatellite markers described by Huang et al.^42^ (Supplementary Table S2). All forward primers were labeled with four kinds of 5′-fluorescein bases (FAM, HEX, TRAMA, or ROX). The multiplex PCR procedure derived from the method of Huang et al.^42^ was run on a BIO-RAD T100^TM^ Thermal Cycler. Detection of multiplex PCR products was carried out with an ABI 3730xl DNA Analyzer by Sangon Biotech Co., Ltd., setting a default range of standard length ± 40 bp. The detection bands of 30 markers were scored using GeneMarker version 1.91 (size standard: GS500).

### Statistical analysis

We employed MICRO-CHECKER 2.2 to detect null alleles, scoring errors, and allele dropout^43^. Linkage disequilibrium and deviation from Hardy–Weinberg equilibrium were tested in Arlequin v. 3.5^44^. No scoring errors or allele dropout was detected by MICRO-CHECKER. Five of 30 loci were null alleles, and no pattern of linkage disequilibrium was found among any loci (*P* > 0.05) (Supplementary Table S3).

Genetic diversity indices, including the number of alleles (*A*), effective number of alleles (*Ae*), observed heterozygosity (*Ho*), expected heterozygosity (*He*), and fixation index (*F*_*IS*_), were calculated for the 278 plants sampled from 19 populations by GenAlEx version 6.503^45^. Analysis of variance (ANOVA) was employed to investigate the influence of isolation on genetic diversity, with Tukey’s HSD test and 95% confidence intervals, by using SPSS^46^.

We separately calculated genetic differentiation and gene flow at the broad and fine scales using GenAlEx^45^. The genetic differentiation coefficient (*F*_*ST*_) was calculated by analysis of molecular variance (AMOVA)^47^. The significance of *F*_*ST*_ values was tested with 999 permutations, followed by Bonferroni correction. Gene flow (*Nm*) was estimated based on the formula $Nm = \left( {1 - F_{ST}} \right)/4F_{ST}$. Hierarchical AMOVA with 999 permutations was constructed to determine the genetic differentiation between populations by GenAlEx^45,47^.

We performed a Mantel test to detect the correlation between the genetic differentiation matrix and geographic distance matrix, using 999 permutations, *F*_*ST*_/(1 − *F*_*ST*_) and logarithmic geographic distance^48^. Since oceanic isolation is the main reason for the genetic divergence of the ZSA population from the mountain populations, we excluded the ZSA population from the Mantel test to remove the interference of oceanic isolation on IBD analysis. The pairwise matrix of geographic distances was calculated based on geographic coordinates, which were identified by a global positioning system (GPS) device (Garmin Oregon 450). All calculations above were performed by GenAlEx^45^.

To infer population structure, Bayesian clustering analysis was conducted in STRUCTURE version 2.3.4^49^. We set the number of groups (*K*) from 1 to 19 with ten independent runs using an admixture ancestry model and 100,000-step Markov chain Monte Carlo (MCMC) replicates after a 10,000-step burn-in for each run. The best *K* value was inferred by delta *K* in STRUCTURE HARVESTER^50,51^. A cluster analysis based on genetic distance was performed by the unweighted pair-group method with arithmetic mean (UPGMA) approach in MEGA 6.0^52^. We used the TREEMIX program to detect historical migration among populations^53^.

BOTTLENECK was used to examine recent bottleneck events^54^ under the TPM (two-phase model), which allows multiple-step mutations^55^, with a 10% infinite allele mutation and 90% stepwise mutation model and 1000 replicates^56^. The significance of heterozygote excess was determined by Wilcoxon’s signed-rank test (*P* < 0.05). Departures from mutation-drift equilibrium were detected by a mode-shift test^57^.

We applied the program DIYABC to infer the divergence history of *A. chinensis* populations^58^. According to the phylogenetic relationships reconstructed by STRUCTURE and TREEMIX, we set a demographic scenario to infer the effective population size and divergence time of four clusters, i.e., TIL islands, neighboring land populations, mountain populations, and the ZSA population. We set the number of simulated data sets to 100,000, and the number of selected data sets to 1000. The historical model, genetic data, and summary statistics were set to default values.

To identify the relationships between genetic diversity and population size in *A. chinensis* and features of TIL islands (Supplementary Table S1), we conducted linear regression analysis with the Pearson correlation coefficient^46^. Features of 11 TIL islands, including area, perimeter, the shape index (SI), distance to the nearest island (DTI), and distance to the nearest mainland (DTL), were measured with a geographic information system (GIS). The island-shape index (SI) was calculated as follows: $SI = P/\left[ {2 \times \left( {\pi \times A} \right)^{0.5}} \right]$^59^.

## Results

### Habitat fragmentation due to geographic isolation on a broad scale

*Actinidia chinensis* plants in the TPZ, XHZ, SYH, YAC, and TIL-M populations were diploid, while 18 plants in the ZSA population were tetraploid (Supplementary Fig. S1). The genetic diversity of *A. chinensis* in TPZ, XHZ, and TIL-M was significantly greater than that in YAC, SYH, and ZSA, and the ZSA population significantly differed from the TPZ, XHZ, and TIL-M populations but not from YAC and SYH (Table 1).

**Table 1:** Genetic characteristics of *A. chinensis* in mountain populations and one oceanic island population (ZSA)

Regions	Altitude (m)	Sample size*	Latitude	Longitude	*A* (SD)	*Ae* (SD)	*Ho* (SD)	*He* (SD)	*F*_*IS*_ (SD)
*Mountain populations*									
TPZ	1143	15	33.6336	111.7094	11.967 (0.710)a	9.302 (0.534)a	0.259 (0.038)b	0.881 (0.007)a	0.711 (0.042)ab
XHZ	617	15	33.5544	110.8840	10.200 (0.624)ab	8.734 (0.556)ab	0.252 (0.042)b	0.871 (0.009)ab	0.715 (0.046)ab
YAC	480	15	25.8001	110.3239	7.833 (0.494)bc	6.719 (0.424)bcd	0.229 (0.047)b	0.837 (0.009)abc	0.731 (0.053)ab
SYH	402	15	30.0398	110.9263	7.600 (0.524)c	6.494 (0.428)cd	0.129 (0.043)b	0.810 (0.029)bc	0.847 (0.049)a
TIL-M^#^	116	15	29.1961	118.6379	12.600 (4.492)a	8.349 (4.010)abc	0.785 (0.196)a	0.839 (0.102)abc	0.068 (0.207)c
Mean					10.040 (4.035)	7.920 (3.158)	0.331 (0.322)	0.848 (0.094)	0.613 (0.373)
*Zhoushan Archipelago population*									
ZSA	108	15	30.0859	122.1157	6.367 (0.334)c	5.289 (0.320)d	0.301 (0.053)b	0.783 (0.018)c	0.619 (0.066)b
Comparisons between mountain populations and ZSA (*P* value)					<0.001	<0.001	0.632	0.001	0.93

*The 15 individuals were randomly selected from all samples to avoid bias due to sample size in calculating genetic diversity

^#^TIL-M, from three neighboring mainland populations in the TIL region, was regarded as an inland population

Different letters in the same column indicate significant differences at *P* < 0.05

*A* number of alleles, *Ae* effective number of alleles, *Ho* observed heterozygosity, *He* expected heterozygosity, *F*_*IS*_ fixation index

In the TPZ, XHZ, SYH, YAC, and ZSA populations, >50% of the tested loci deviated from Hardy–Weinberg equilibrium (Supplementary Table S4), indicating frequent inbreeding. Mountain populations (XHZ, TPZ, SYH, and YAC) showed significant heterozygote excess but no evidence of bottleneck events (Supplementary Table S5), while the ZSA population experienced recent bottleneck events according to departures from mutation-drift equilibrium in the allele frequency distribution (Supplementary Table S5).

**Fig. 2:**
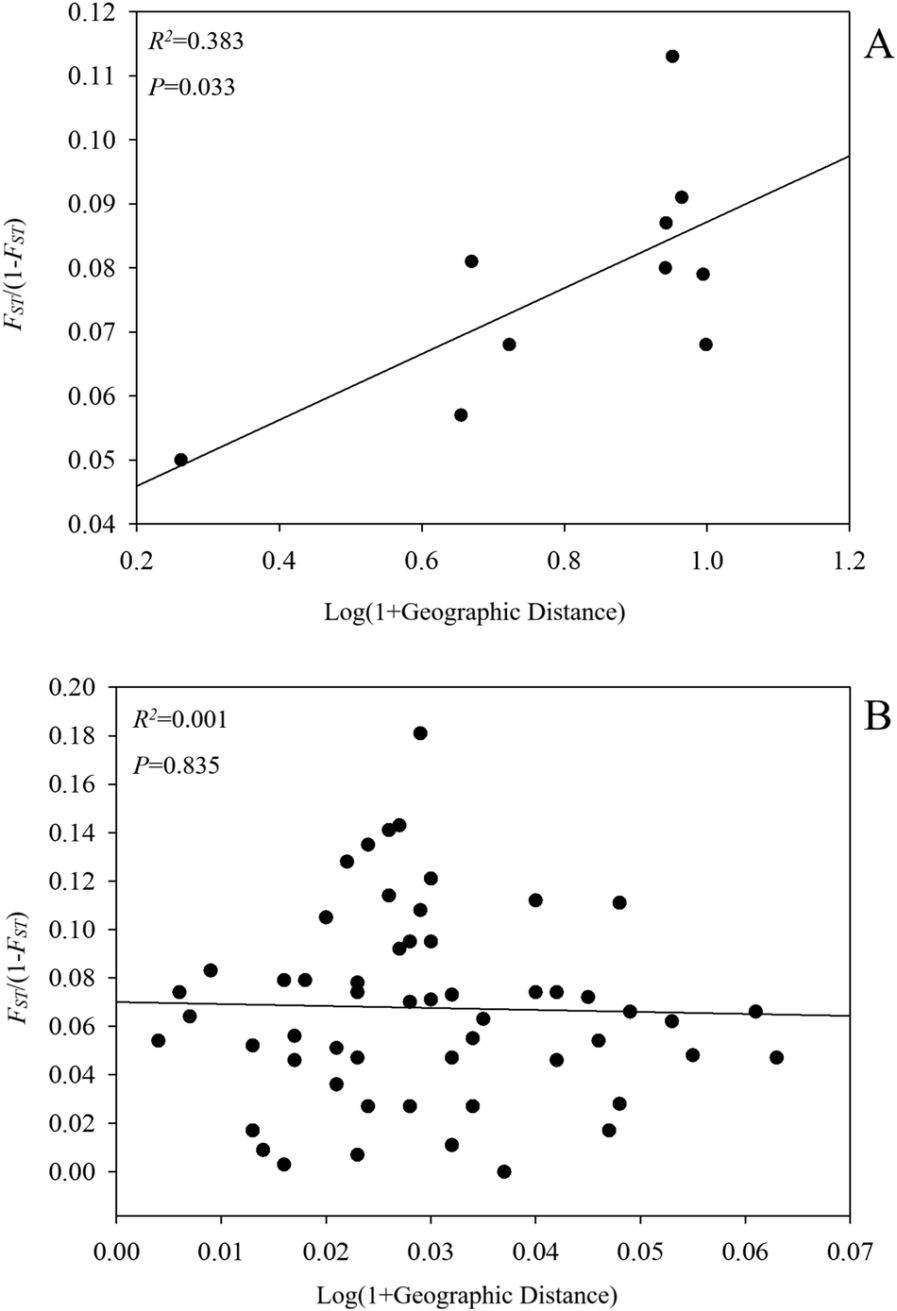
The relationship between genetic differentiation and geographical distance in *A. chinensis* populations. Genetic differentiation was calculated as [*F*_*ST*_/(1 − *F*_*ST*_)], and geographical distance was log-transformed. A, five mountain populations on a broad scale; B, TIL populations

The pairwise-estimated *F*_*ST*_ between populations was moderate (0.012–0.123), with the lowest *F*_*ST*_ index in the comparison of ZSA and SYH populations (Table 2). The gene flow (*Nm*) between populations ranged from 1.786 to 20.833, with the highest *Nm* in the comparison of the ZSA and SYH populations (Table 2). The Mantel test showed that genetic differentiation was positively related to geographical distance (log-transformed) on a broad scale (*R*^2^ = 0.383, *P* = 0.033) (Fig. 2), indicating an isolation-by-distance effect.

**Table 2:** Matrix of pairwise *F*_*ST*_ values and *Nm* coefficients of six *A. chinensis* populations on the broad scale

TIL-M	TPZ	XHZ	SYH	YAC	ZSA	
	0.074	0.064	0.053	0.064	0.083	TIL-M
3.130		0.021	0.076	0.048	0.123	TPZ
3.685	11.522		0.053	0.026	0.092	XHZ
4.467	3.022	4.486		0.034	0.012	SYH
3.681	4.974	9.524	7.028		0.066	YAC
2.763	1.786	2.475	20.833	3.539		ZSA

Values above the diagonal are pairwise genetic differentiation coefficients, *F*_*ST*_. Values below the diagonal are pairwise gene-flow coefficients, *Nm*

**Fig. 3:**
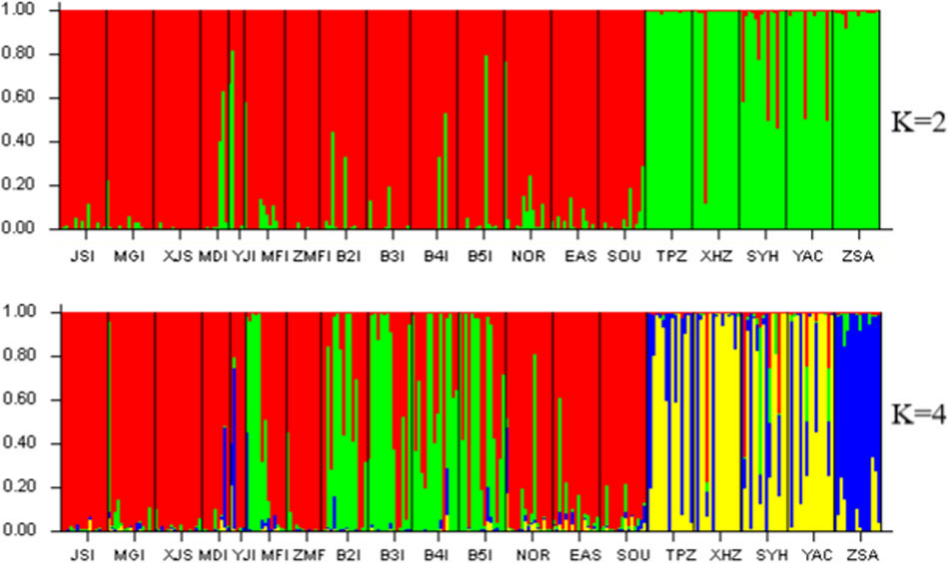
Genetic structure of *A. chinensis* populations. Population structure bar plots show the clustering of samples into two (best delta *K*) and four clusters. Each vertical bar indicates an individual, and the height of each colored bar represents the proportion of assignment to that cluster. Abbreviations are population names; see Fig. 1

Structure analysis showed that the ZSA population differed from other mainland populations (*K* = 4) (Fig. 3), and UPGMA cluster analysis confirmed these structure results (Supplementary Fig. S2). Analysis of molecular variance (AMOVA) was performed between ZSA and the mountain populations, and only 3% (*P* < 0.05) of the total genetic variance was attributed to the differences between the two groups. The vast majority (90%, *P* < 0.05) of the variance occurred among individuals within populations, whereas only 7% of variance occurred among populations (Supplementary Table S6).

**Fig. 4:**
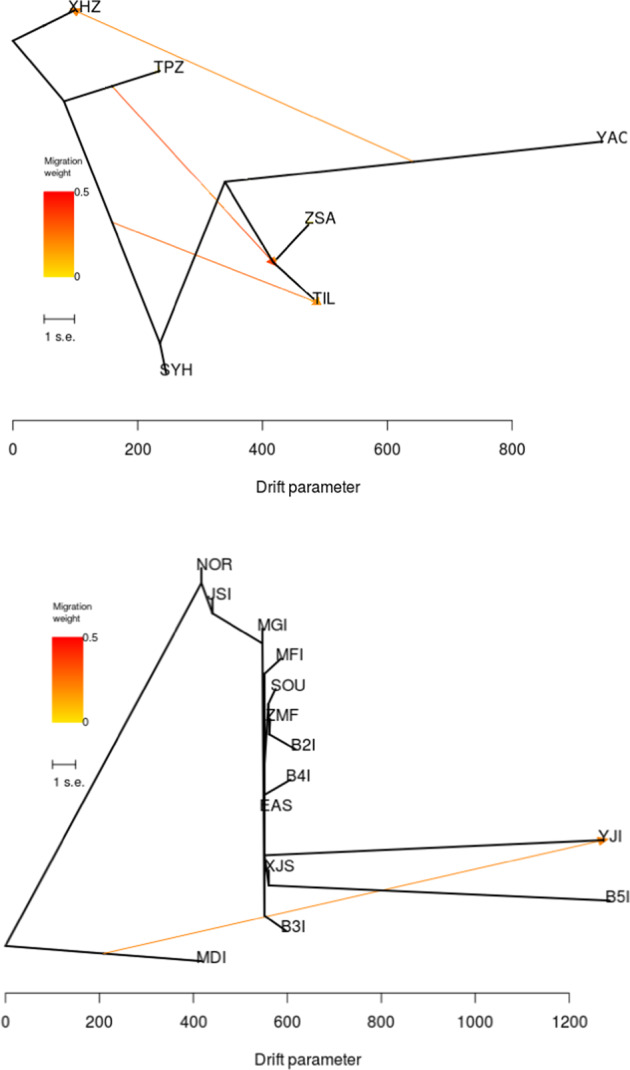
The historical mitigation between the *A. chinensis* populations. Top subfigure, *A. chinensis* populations at the broad scale; bottom subfigure, the *A. chinensis* TIL populations at the fine scale

Approximate Bayesian Computation (DIYABC) revealed a smaller effective population size (*Ne*) in ZSA than in the mountain populations (Supplementary Table S7 and Supplementary Fig. S3). The differentiation time of the ZSA population (median *t*2 = 1920 generations ≈11,520 years, mode *t*2 = 1290 generations ≈7740 years) coincided with the formation of ZSA islands (ca. 7000–9000 years ago). The ZSA population differentiated a mean of 2450 generations ago, later than the formation time of the mountain populations (8880 generations; Supplementary Table S7). TREEMIX analysis revealed historic migration from the northern TPZ population to the eastern TIL-M and ZSA populations, but not to the southern SYH and YAC populations (Fig. 4).

### Habitat fragmentation from water isolation on a fine scale in TIL

All 480 sampled plants in TIL were diploid (Supplementary Fig. S1). The population size of *A. chinensis* per island was positively correlated with island area (*r* = 0.963, *P* < 0.001) and perimeter (*r* = 0.943, *P* < 0.001) (Supplementary Tables S1 and S8). More than one-half of the *A. chinensis* plants were seedlings on the XJS, B3I, and B4I islands, and most of the seedlings appeared at the edge of the islands.

With a hypothesis of plants migrating from old to young populations, we can detect possible dispersal pathways of *A. chinensis* by measuring tree rings to define the age of plants in populations. Linear regression analysis revealed a significant positive correlation between tree rings and DBH (Supplementary Fig. S4) (*R*^2 ^= 0.669, *P* = 0.023). The maximum DBH of sampled *A. chinensis* plants on the TIL islands was 30.8 cm in the MGI population (Supplementary Table S1), indicating that the oldest plant was ~23 years old. The maximum DBHs of plants on TIL islands were similar and did not show dispersal pathways, indicating the simultaneous existence of *A. chinensis* plants on these islands. However, the TREEMIX results showed historic migration from MDI to YJI islands (Fig. 4). The differentiation time between TIL island populations and neighboring mainland populations was 62 generations (6 years/generation; Supplementary Table S7), indicating that the present *A. chinensis* plants emerged before the formation of the TIL reservoir (ca. 60 years ago).

The genetic diversity of *A. chinensis* was not related to TIL island features or geographic distance between islands (Supplementary Table S8). The genetic diversity of *A. chinensis* populations in the neighboring mainland populations and on large islands (JSI and MGI) was higher than that on other small islands (Table 3). The absolute value of *F*_*IS*_ of all populations was close to zero (Table 3), indicating random mating and a limited isolation effect in these populations. The TIL populations showed significantly higher *Ho* and lower *F*_*IS*_ values than the other populations (Table 1).

**Table 3:** Genetic characteristics of *A. chinensis* populations on 11 islands and in three neighboring mainland populations in TIL

Populations	Sample size*	Latitude	Longitude	*A* (SD)	*Ae* (SD)	*Ho* (SD)	*He* (SD)	*F*_*IS*_ (SD)
*Islands*								
JSI	15	29.5234	118.8831	10.800 (0.526)a	7.541 (0.515)a	0.739 (0.038)ab	0.843 (0.014)a	0.128 (0.045)ab
MGI	15	29.5272	118.8534	10.900 (0.586)a	7.642 (0.563)a	0.769 (0.039)ab	0.835 (0.020)ab	0.088 (0.042)abc
XJS	15	29.6331	118.8919	8.967 (0.633)ab	6.258 (0.491)abc	0.709 (0.046)ab	0.782 (0.034ab	0.083 (0.050)abc
MDI	9	29.5718	118.8847	6.433 (0.527)bc	4.788 (0.480)bc	0.742 (0.043)ab	0.718 (0.033)bc	−0.043 (0.045)bc
YJI	5	29.5635	118.8834	4.567 (0.476)c	3.963 (0.469)c	0.721 (0.061)ab	0.635 (0.043)c	−0.152 (0.083)c
MFI	13	29.5783	118.8967	11.400 (0.513)a	8.198 (0.514)a	0.755 (0.027)ab	0.861 (0.010)a	0.125 (0.029)ab
ZMF	11	29.5868	118.9355	9.867 (0.621)a	7.158 (0.594)ab	0.849 (0.025)a	0.821 (0.018)ab	−0.039 (0.028)bc
B2I	15	29.5017	118.8097	10.233 (0.671)a	6.951 (0.585)ab	0.700 (0.048)ab	0.822 (0.016)ab	0.152 (0.058)ab
B3I	15	29.5126	118.8463	9.233 (0.666)ab	6.449 (0.503)abc	0.643 (0.058)ab	0.810 (0.017)ab	0.213 (0.072)a
B4I	15	29.4971	118.8981	10.400 (0.624)a	6.972 (0.514)ab	0.669 (0.045)ab	0.823 (0.018)ab	0.199 (0.047)ab
B5I	15	29.5173	118.9238	8.933 (0.826)ab	5.976 (0.640)abc	0.630 (0.053)b	0.744 (0.038)abc	0.154 (0.066)ab
*Neighboring mainland populations (TIL-M)*								
NOR	15	29.5764	118.8527	11.833 (0.671)a	8.477 (0.701)a	0.766 (0.038)ab	0.844 (0.019)a	0.097 (0.041)abc
EAS	15	29.4301	118.938	9.433 (0.550)a	6.752 (0.480)ab	0.784 (0.038)ab	0.812 (0.023)ab	0.037 (0.039)abc
SOU	15	29.1961	118.6379	9.000 (0.786)ab	6.390 (0.660)abc	0.775 (0.042)ab	0.782 (0.024)ab	0.007 (0.061)abc
*Comparison between island and mainland populations*								
Mean values on islands				9.248 (3.850)	6.536 (3.130)	0.721 (0.251)	0.790 (0.154)	0.084 (0.308)
Mean values on the mainland				10.089 (3.871)	7.206 (3.485)	0.775 (0.214)	0.813 (0.123)	0.047 (0.263)
*P* value				0.067	0.080	0.061	0.209	0.306

*The 15 individuals were randomly selected from all samples to avoid bias from sample size in calculating genetic diversity

Different letters in the same column indicate significant differences at *P* < 0.05

*A* number of alleles, *Ae* effective number of alleles, *Ho* observed heterozygosity, *He* expected heterozygosity, *F*_*IS*_ fixation index

The pairwise-estimated *F*_*ST*_ between populations in TIL ranged from 0 to 0.163, with a high level of gene flow between these populations (*Nm* from 1.285 to 82.245) (Supplementary Table S9). Recent bottleneck events were not detected in TIL island populations, except for the YJI population with a small population size and small island area (Supplementary Table S5). Structure analysis revealed that the TIL populations differed from other mountain populations (*K* = 2), and the nearby B2I, B3I, B4I, and B5I islands exhibited frequent gene flow (Fig. 3).

The AMOVA results attributed a low percentage of the total genetic variance (1%) to differences between TIL island and neighboring land populations, with 5% among populations and 94% within populations (Supplementary Table S6). Genetic differentiation of island populations was not related to geographic distance between islands (*R*^2 ^= 0.001, *P* = 0.835) (Fig. 2).

## Discussion

Habitat fragmentation has been found to reduce genetic diversity^56,60,61^, while some studies have found no negative effects^21,22^. To investigate whether the differentiated genetic effects of habitat fragmentation are associated with the scale of habitat fragmentation, we measured the genetic diversity of *A. chinensis* populations at a broad and a fine scale. Our results showed scale-dependent effects of habitat fragmentation on *A. chinensis* populations, with an isolation-by-distance effect at the broad scale, but no negative effects on genetic diversity at the TIL (fine) scale.

The positive correlation between *A. chinensis* population size and island area in TIL supported island biogeography theory^62–64^, while the relationship between the genetic diversity of *A. chinensis* and island area did not. Landscape attributes are key factors determining the genetic diversity effects of habitat fragmentation^65–67^. Small habitat area and island isolation have been found to decrease the genetic diversity of plant species^68^. Landscape connectivity is beneficial to the conservation of genetic diversity and helps reduce demographic bottlenecks in natural populations^66^. We found most seedlings on the edges of TIL islands, and the ripe fruits of *A. chinensis* are able to float in freshwater. The upstream Xin’an River is located west of the TIL and leads to northern and southern flow from west to east, which likely explains the northern and southern clusters (Fig. 3 and Supplementary Fig. S2). The results showed low pairwise-estimated *F*_*ST*_, low *F*_*IS*_, and no bottleneck events (except in YJI). These results indicate that seed dispersal with lake water flow facilitates gene flow between islands.

In addition, the mating pattern, dioecy, and relatively long lifespan of *A. chinensis* may buffer the area, isolation, and edge effects resulting from the fine scale of habitat fragmentation in TIL^29,69,70^. Dioecy can decrease the inbreeding rate and counteract the negative effects of isolation due to forced outcrossing^71^. Dioecious species experience lower frequencies of genetic drift than monoecious species^28^. Species with short lifespans in highly fragmented landscapes may experience genetic degradation due to the loss and fixation of alleles^29^, while long-lived species could resist the genetic erosion caused by habitat fragmentation^29,60^. The age of *A. chinensis* plants was greater than 20 years (Supplementary Fig. S4), and *A. chinensis* plants almost simultaneously emerged on these islands before the formation of the TIL reservoir (Supplementary Table S7), except for individual plants in the YJI island population that migrated from the MDI island (Fig. 4).

However, the buffering mechanisms discussed above were limited at the broad scale because geographic isolation resulted in low gene flow and high differentiation between mountain populations, and led to an isolation-by-distance effect. This was consistent with the findings of other studies on a broad scale of habitat fragmentation^26,30^. These mountain populations are located in isolated island-like habitats that lack connectivity between populations, which limits seed dispersal and insect pollination. Inbreeding caused by isolation leads to the fixation and loss of alleles and then the loss of genetic variance^17,18^. We found loci deviating from HWE and high fixation indices (*F*_*IS*_ > 0.7) in ZSA island and mountain populations.

The ZSA population, on an oceanic island, significantly differed from the mainland populations (Fig. 3 and Supplementary Fig. S3), with high *F*_*IS*_ values and bottleneck events (Table 1 and Supplementary Table S5). Founder effects could not explain this phenomenon. The divergence time between mainland and the ZSA population coincides with the formation time of ZSA, but ZSA is genetically closer to the far-away SYH population than to the nearby TIL population. The results showed high gene flow and low differentiation between the ZSA and SYH populations, resulting in similar levels of genetic diversity between them. The Yangtze River connects the two populations, which might facilitate the seed dispersal of *A. chinensis*. As discussed above, in TIL, seed dispersal via floating on water is the main dispersal method of *A. chinensis*. Gene flow through long-distance pollen or seed dispersal could disrupt isolation and weaken the negative effects of habitat fragmentation^15,16,69^. In fact, some studies revealed gene flow and introgression between diploid and tetraploid populations of *A. chinensis*^72^. Breeding experiments showed that diploid *A. chinensis* could crossbreed with tetraploid *A. chinensis* and produce fertile offspring^73^. The existence of transitional phenotypes of *A. chinensis* in the wild proves the occurrence of gene flow and introgression between the two cytotypes in nature^74^. This phenomenon suggests that the design of corridors is very important for the conservation of genetic diversity.

*A. chinensis* plants in ZSA were tetraploid, and their origin was unclear. Diploid *A. chinensis* is thought to be the progenitor of tetraploid *A. chinensis*^75,76^. We detected historical migration events from TPZ to ZSA, indicating that the ZSA population may have come from northern populations, but the mechanism of polyploidization is still unknown. Generally, polyploidization can increase biological diversity, but the genetic diversity of the tetraploid ZSA population was lower than that of the diploid populations. This could be explained by treating tetraploid plants as diploid plants to score bands, the high inbreeding rate of the ZSA population, and bottleneck events resulting from the small population size. Llorens et al. reported bottleneck events and decreased genetic diversity in the shrubs *Grevillea caleyi* and *G. longifolia*, indicating that they had experienced long-term habitat isolation^77^. Moreover, the detrimental effects of long-term local adaptation likely decrease genetic diversity^78^. ZSA is an oceanic island that has undergone long-term isolation of ca. 7000–9000 years^27^. Long-term oceanic isolation decreased the genetic diversity and increased the genetic differentiation of insular populations, while there were small genetic effects on the TIL islands that experienced short-term isolation by water. This is likely to support a time-delayed effect of habitat fragmentation. Such time delays tend to mask the genetic effects of habitat fragmentation^79^.

In conclusion, we found a scale-dependent effect of habitat fragmentation on the genetic diversity of *A. chinensis* populations, with an isolation-by-distance effect at the broad scale. The connectivity between populations explains the scale-dependent effect because it facilitates the seed dispersal of *A. chinensis* and buffers against the negative effects of habitat fragmentation, not only at the fine scale but also at the broad scale. Therefore, our results highlight the importance of constructing corridors for biodiversity conservation, particularly in broad-scale island-like fragmented habitats and on long-term isolated islands.

## Supplementary information

Supplementary materials
