# Supplementary material for: Scale-dependent effects of habitat fragmentation on the genetic diversity of Actinidia chinensis populations in China
Source: Hortic Res. 2020 Oct 13;7:172. doi: 10.1038/s41438-020-00401-1 (PMC7553913; doi:10.1038/s41438-020-00401-1)
Supplement: Supplementary file 1 — Supplementary materials [file 41438_2020_401_MOESM1_ESM.docx]

Scale-dependent effects of habitat fragmentation on the genetic diversity of *Actinidia chinensis* populations in China

Wenhao Yu^1^, Baofeng Wu^1^, Xinyu Wang^1^, Zhi Yao^1^, Yonghua Li^1^ and Yongbo Liu^1,*^

^1^ State Key Laboratory of Environmental Criteria and Risk Assessment, Chinese Research Academy of Environmental Sciences, 8 Dayangfang, Beijing 100012, China

*^*^Corresponding author*:

State Key Laboratory of Environmental Criteria and Risk Assessment,

Chinese Research Academy of Environmental Sciences

8 Dayangfang, Beijing 100012, China

Tel/Fax: 86-10-84910906;

E-mails: [liu.yongbo@yahoo.com](mailto:liu.yongbo@yahoo.com); [liuyb@craes.org.cn](mailto:liuyb@craes.org.cn)

**Table S1** Population characteristics of *A. chinensis* and island features in the TIL region

| Island | | Population characteristics of *A. chinensis* | | | |  | Island features in the TIL | | | | |
| --- | --- | --- | --- | --- | --- | --- | --- | --- | --- | --- | --- |
|  | | Population size | Max.of DBH (cm) | No. of Adults | No. of Seedlings |  | Area  (km^2^) | Perimeter  (km) | SI | DTI  (m) | DTL  (m) |
| JSI | 561 (188)^†^ | 24.8 | 116 | 71 |  | 13.515 | 54.255 | 4.014 | 81 | 844 |  |
| MGI | 315 (135)^†^ | 30.8 | 75 | 60 |  | 8.599 | 44.896 | 5.221 | 81 | 646 |  |
| XJS | 100 | 22 | 34 | 66 |  | 1.822 | 10.317 | 5.662 | 2140 | 101 |  |
| B2I | 17 | 25.8 | 17 | 0 |  | 1.676 | 9.087 | 5.421 | 803 | 1469 |  |
| ZMF | 14 | 7.5 | 1 | 13 |  | 1.517 | 11.569 | 7.626 | 2140 | 88 |  |
| B5I | 18 | 17.2 | 11 | 2 |  | 1.192 | 7.623 | 6.395 | 154 | 916 |  |
| YJI | 5 | 12.5 | 1 | 4 |  | 0.862 | 5.644 | 6.547 | 51 | 713 |  |
| MDI | 9 | 21.7 | 9 | 0 |  | 0.444 | 3.695 | 8.327 | 51 | 65 |  |
| B3I | 126 | 29.8 | 51 | 75 |  | 0.436 | 4.259 | 9.765 | 93 | 2194 |  |
| MFI | 13 | 21.8 | 7 | 6 |  | 0.188 | 2.35 | 12.471 | 774 | 49 |  |
| B4I | 47 | 19.5 | 11 | 36 |  | 0.068 | 6.367 | 93.094 | 494 | 97 |  |

^†^ The population size was estimated, number in brackets indicating the number of sampled plants

Abbreviations: SI, $shape index=P/\left[ 2\times\left( \pi\times A \right)^{0.5} \right]$; DTI, distance to the nearest island; DTL, distance to the nearest mainland; DBH, diameter of breast height of *A. chinensis* plants

**Table S2** Primer sequence, product sizes, fluorescent and melting temperature (Tm) of 30 microsatellite markers

| Locus code | Primer sequences (5’-3’) | Standard length(bp) | Tm | Fluorescent |
| --- | --- | --- | --- | --- |
| UDK96-001 | GAATCGCGTAATGATTGATGG | 258 | 55 | FAM |
|  | GTTTCCCACTCTGCAAAAGC |  |  |  |
| UDK96-009 | CACTCACATGCCTTTACACACA | 172 | 55 | FAM |
|  | AAGAGGCCACCAAAAACCTT |  |  |  |
| UDK96-013 | ACGTGACTTGGTTTTTGAAGG | 132 | 55 | FAM |
|  | CACTCCGATCAGCTCTCCTC |  |  |  |
| UDK96-016 | TAGGTGAAAGACACACCACAC | 199 | 57 | FAM |
|  | ATAAACGTCATGGGCTCAGC |  |  |  |
| UDK96-017 | CCCGTGAGATGTAACGTCAC | 162 | 55 | HEX |
|  | TGAGAGAGAGAGTGGAGCTCG |  |  |  |
| UDK96-018 | GTAAACTGCATTTGGTCCTCG | 164 | 55 | FAM |
|  | TTAGTTTTCATCCTTGGTTCCA |  |  |  |
| UDK96-019 | ATACACTTGAAGCGCCGC | 149 | 55 | HEX |
|  | AAGCAGCCATGTCGATACG |  |  |  |
| UDK96-022 | GGCGTCAAGGTTCTCTCTCT | 85 | 55 | TAMRA |
|  | TGGGGTAACTACTCACTAAGC |  |  |  |
| UDK96-023 | CAAACGAGTCCTAGATCTTGC | 145 | 55 | FAM |
|  | ATCTGGAGCCATACGGAGG |  |  |  |
| UDK96-026 | CGCTGACCAGATTCTGATGA | 173 | 55 | HEX |
|  | TGAAAATCACTGAGCACAACC |  |  |  |
| UDK96-028 | TCCCCACACAACAACTCCTC | 150 | 55 | HEX |
|  | CAGATGACCTGCACGTGC |  |  |  |
| UDK96-030 | TCATGTTTGTGGTTGAGTTGTG | 115 | 55 | FAM |
|  | AGCAATAAACTCAAGCGCGT |  |  |  |
| UDK96-033 | AGGCAGCAATGCTCTCTCTC | 130 | 57 | HEX |
|  | CTGGTGGACAGGGCTAAAG |  |  |  |
| UDK96-034 | TTATATGGTGCGGCATGCTA | 201 | 55 | HEX |
|  | TGAATGCAGAAGGCAATCAG |  |  |  |
| UDK96-037 | CATTTCAGTGTTAACTCTTTCG | 122 | 55 | ROX |
|  | CCCTCTTTCAAAGGTTCTCT |  |  |  |
| UDK96-039 | GGTTTGATCGGTCTTCGAAA | 148 | 57 | TAMRA |
|  | ATAAATGTGTGCCAGTGCGA |  |  |  |
| UDK96-040 | TCGAGTTACCTAGCTACTCCGC | 177 | 55 | TAMRA |
|  | AAGGGAAGAAAATGTTGAACC |  |  |  |
| UDK97-401 | GAGCAAAAAGCTTGACACCC | 146 | 57 | FAM |
|  | ACCTGGTGGTAGATCCTCCA |  |  |  |
| UDK97-402 | ATATGATAGAACGCCATCCCC | 126 | 57 | HEX |
|  | GACGTGCCATCAGTTTCCTT |  |  |  |
| UDK97-404 | CGGCATTTTCTTTTTAATGACC | 151 | 55 | TAMRA |
|  | TTGCCTTGCTCTTGTTCATG |  |  |  |
| UDK97-405 | TACACGGAGTACAAAGGTGGG | 149 | 55 | HEX |
|  | AACCGATGATCAAGAAAACCC |  |  |  |
| UDK97-407 | CGTTCGAGGGTAAGGAGTTG | 110 | 55 | TAMRA |
|  | CCTAACCCGACCGTAGTGTG |  |  |  |
| UDK97-408 | GTGCTCCTCCGTCCATGTAT | 111 | 55 | ROX |
|  | CGTCCTCTCTTCGCCATTTA |  |  |  |
| UDK97-411 | TGATTAAATCCCCAAACCCA | 148 | 55 | TAMRA |
|  | TCCCAATCTCATCATCCCTC |  |  |  |
| UDK97-412 | CAAACTCGAACCACATTAACA | 100 | 55 | TAMRA |
|  | TAGCCTGTTGCCGGCTAG |  |  |  |
| UDK97-415 | TCTTCAAATGGGCATGTTGA | 145 | 55 | ROX |
|  | CCAGCCAATCTCTCCCAATA |  |  |  |
| UDK97-419 | TGGGTAGTTTCTCTGGTTTTGC | 131 | 55 | ROX |
|  | CATGGGGTTTTCTAAAGACTGC |  |  |  |
| UDK97-420 | GAGCGTAGAATGTGGCGG | 136 | 57 | TAMRA |
|  | ATTTGAAATGAGTCAAGCTGC |  |  |  |
| UDK97-421 | AATCGGACGTCGCTTCTG | 117 | 55 | ROX |
|  | TCGTTGGGTCTCCAAATCTC |  |  |  |
| UDK97-422 | GGAGAGGTGATTGTGGCAGT | 149 | 55 | ROX |
|  | CTTATCATCTGCGCCCTCTC |  |  |  |

**Table S3** Testing of linkage disequilibrium among 30 microsatellite loci (significance level=0.05)

| locus | 1 | 2 | 3 | 4 | 5 | 6 | 7 | 8 | 9 | 10 | 11 | 12 | 13 | 14 | 15 | 16 | 17 | 18 | 19 | 20 | 21 | 22 | 23 | 24 | 25 | 26 | 27 | 28 | 29 | 30 |
| --- | --- | --- | --- | --- | --- | --- | --- | --- | --- | --- | --- | --- | --- | --- | --- | --- | --- | --- | --- | --- | --- | --- | --- | --- | --- | --- | --- | --- | --- | --- |
| 1 | * | + | + | + | + | + | + | + | + | + | + | + | + | + | + | + | + | + | + | + | + | + | + | + | + | + | + | + | + | + |
| 2 | + | * | + | + | + | + | + | + | + | + | + | + | + | + | + | + | + | + | + | + | + | + | + | + | + | + | + | + | + | + |
| 3 | + | + | * | + | + | + | + | + | + | + | + | + | + | + | + | + | + | + | + | + | + | + | + | + | + | + | + | + | + | + |
| 4 | + | + | + | * | + | + | + | + | + | + | + | + | + | + | + | + | + | + | + | + | + | + | + | + | + | + | + | + | + | + |
| 5 | + | + | + | + | * | + | + | + | + | + | + | + | + | + | + | + | + | + | + | + | + | + | + | + | + | + | + | + | + | + |
| 6 | + | + | + | + | + | * | + | + | + | + | + | + | + | + | + | + | + | + | + | + | + | + | + | + | + | + | + | + | + | + |
| 7 | + | + | + | + | + | + | * | + | + | + | + | + | + | + | + | + | + | + | + | + | + | + | + | + | + | + | + | + | + | + |
| 8 | + | + | + | + | + | + | + | * | + | + | + | + | + | + | + | + | + | + | + | + | + | + | + | + | + | + | + | + | + | + |
| 9 | + | + | + | + | + | + | + | + | * | + | + | + | + | + | + | + | + | + | + | + | + | + | + | + | + | + | + | + | + | + |
| 10 | + | + | + | + | + | + | + | + | + | * | + | + | + | + | + | + | + | + | + | + | + | + | + | + | + | + | + | + | + | + |
| 11 | + | + | + | + | + | + | + | + | + | + | * | + | + | + | + | + | + | + | + | + | + | + | + | + | + | + | + | + | + | + |
| 12 | + | + | + | + | + | + | + | + | + | + | + | * | + | + | + | + | + | + | + | + | + | + | + | + | + | + | + | + | + | + |
| 13 | + | + | + | + | + | + | + | + | + | + | + | + | * | + | + | + | + | + | + | + | + | + | + | + | + | + | + | + | + | + |
| 14 | + | + | + | + | + | + | + | + | + | + | + | + | + | * | + | + | + | + | + | + | + | + | + | + | + | + | + | + | + | + |
| 15 | + | + | + | + | + | + | + | + | + | + | + | + | + | + | * | + | + | + | + | + | + | + | + | + | + | + | + | + | + | + |
| 16 | + | + | + | + | + | + | + | + | + | + | + | + | + | + | + | * | + | + | + | + | + | + | + | + | + | + | + | + | + | + |
| 17 | + | + | + | + | + | + | + | + | + | + | + | + | + | + | + | + | * | + | + | + | + | + | + | + | + | + | + | + | + | + |
| 18 | + | + | + | + | + | + | + | + | + | + | + | + | + | + | + | + | + | * | + | + | + | + | + | + | + | + | + | + | + | + |
| 19 | + | + | + | + | + | + | + | + | + | + | + | + | + | + | + | + | + | + | * | + | + | + | + | + | + | + | + | + | + | + |
| 20 | + | + | + | + | + | + | + | + | + | + | + | + | + | + | + | + | + | + | + | * | + | + | + | + | + | + | + | + | + | + |
| 21 | + | + | + | + | + | + | + | + | + | + | + | + | + | + | + | + | + | + | + | + | * | + | + | + | + | + | + | + | + | + |
| 22 | + | + | + | + | + | + | + | + | + | + | + | + | + | + | + | + | + | + | + | + | + | * | + | + | + | + | + | + | + | + |
| 23 | + | + | + | + | + | + | + | + | + | + | + | + | + | + | + | + | + | + | + | + | + | + | * | + | + | + | + | + | + | + |
| 24 | + | + | + | + | + | + | + | + | + | + | + | + | + | + | + | + | + | + | + | + | + | + | + | * | + | + | + | + | + | + |
| 25 | + | + | + | + | + | + | + | + | + | + | + | + | + | + | + | + | + | + | + | + | + | + | + | + | * | + | + | + | + | + |
| 26 | + | + | + | + | + | + | + | + | + | + | + | + | + | + | + | + | + | + | + | + | + | + | + | + | + | * | + | + | + | + |
| 27 | + | + | + | + | + | + | + | + | + | + | + | + | + | + | + | + | + | + | + | + | + | + | + | + | + | + | * | + | + | + |
| 28 | + | + | + | + | + | + | + | + | + | + | + | + | + | + | + | + | + | + | + | + | + | + | + | + | + | + | + | * | + | + |
| 29 | + | + | + | + | + | + | + | + | + | + | + | + | + | + | + | + | + | + | + | + | + | + | + | + | + | + | + | + | * | + |
| 30 | + | + | + | + | + | + | + | + | + | + | + | + | + | + | + | + | + | + | + | + | + | + | + | + | + | + | + | + | + | * |

The symbol “+” means no significant linkage disequilibrium between loci

**Table S4** Testing of departure from Hardy-Weinberg equilibrium (significance level=0.05)

| Population | JSI | MGI | XJS | MDI | YJI | MFI | ZMF | B2I | B3I | B4I | B5I | NOR | EAS | SOU | TPZ | XHZ | SYH | YAC | ZSA |
| --- | --- | --- | --- | --- | --- | --- | --- | --- | --- | --- | --- | --- | --- | --- | --- | --- | --- | --- | --- |
| UDK96-001 | + | + | + | - | - | + | - | - | - | + | - | - | - | - | + | + | + | + | + |
| UDK96-009 | - | - | - | - | - | - | - | + | - | - | - | + | - | + | + | + | + | + | + |
| UDK96-013 | + | - | + | - | - | - | - | - | - | - | - | + | - | - | + | + | + | + | - |
| UDK96-016 | - | - | + | - | - | + | - | + | - | + | - | - | - | - | + | + | + | + | + |
| UDK96-017 | + | + | - | - | - | - | - | + | - | + | - | - | + | - | + | + | + | + | - |
| UDK96-018 | + | - | - | - | - | - | - | + | + | + | - | - | - | - | + | + | - | + | - |
| UDK96-019 | + | - | - | + | - | - | - | - | - | - | - | - | - | - | + | + | - | - | - |
| UDK96-022 | + | - | - | - | - | - | - | + | - | - | - | - | - | - | + | + | - | + | - |
| UDK96-023 | - | - | - | - | - | - | - | + | - | - | - | - | - | - | + | + | - | - | - |
| UDK96-026 | - | - | - | + | + | + | - | + | + | + | + | - | - | - | + | + | - | + | - |
| UDK96-028 | + | + | - | + | - | + | - | + | + | + | + | - | + | - | + | + | + | + | - |
| UDK96-030 | - | - | - | - | - | - | - | - | - | - | + | - | - | - | + | + | + | + | - |
| UDK96-033 | + | - | - | - | - | + | - | - | + | + | + | + | - | - | + | + | + | + | - |
| UDK96-034 | - | - | - | - | - | - | - | - | - | - | - | - | - | - | + | + | + | + | - |
| UDK96-037 | - | - | + | - | - | - | - | + | + | - | - | - | - | - | + | + | + | + | - |
| UDK96-039 | - | - | - | - | - | + | - | - | - | - | - | - | - | - | + | + | + | + | - |
| UDK96-040 | - | - | - | - | - | - | - | - | - | - | - | - | - | - | + | + | + | + | + |
| UDK97-401 | - | - | - | - | - | - | - | - | - | - | - | - | - | - | + | - | - | + | + |
| UDK97-402 | + | + | - | - | - | + | - | + | + | + | + | - | - | + | + | + | + | + | + |
| UDK97-404 | - | - | - | - | - | + | - | - | - | - | - | - | - | - | + | + | - | + | + |
| UDK97-405 | - | - | - | - | - | - | - | - | - | - | - | - | + | + | + | - | - | - | - |
| UDK97-407 | - | - | - | - | + | + | - | - | - | - | - | - | - | - | + | + | + | + | + |
| UDK97-408 | - | - | - | - | - | - | + | - | + | + | + | - | - | - | - | - | - | - | - |
| UDK97-411 | - | - | - | - | - | + | - | - | - | - | - | + | - | - | + | + | + | + | + |
| UDK97-412 | - | - | + | - | - | - | - | + | + | - | + | - | - | - | + | + | + | - | + |
| UDK97-415 | - | - | - | - | - | - | - | - | - | - | - | - | - | - | + | - | + | + | + |
| UDK97-419 | + | - | - | - | - | - | - | + | + | - | - | + | - | - | + | + | + | + | + |
| UDK97-420 | - | - | + | - | - | - | - | + | - | + | - | - | + | - | + | + | + | + | + |
| UDK97-421 | - | - | - | - | - | - | - | + | + | - | - | - | - | - | + | + | + | + | + |
| UDK97-422 | + | - | - | - | - | - | - | - | - | - | + | - | - | - | + | - | - | + | + |
| Percentage of loci deviated from HWE | 37% | 13% | 20% | 10% | 7% | 33% | 3% | 47% | 33% | 33% | 27% | 17% | 13% | 10% | 97% | 83% | 67% | 83% | 50% |

The symbol “+” means departure from Hardy-Weinberg equilibrium in the locus

**Table S5** Bottleneck analysis of *A. chinensis* in mountain populations, the ZSA island and TIL islands

| Population | Two-phase mutation model | |
| --- | --- | --- |
|  | Wilcoxon sign rank test | Mode-shift test |
| Mountain populations | | |
| TPZ | ＜0.001 | L-shaped |
| XHZ | ＜0.001 | L-shaped |
| YAC | ＜0.001 | L-shaped |
| SYH | ＜0.001 | L-shaped |
| TIL-M^†^ | 0.672 | L-shaped |
| Zhoushan Archipelago population | | |
| ZSA | ＜0.001 | shifted |
| TIL island populations | | |
| JSI | 0.657 | L-shaped |
| MGI | 0.428 | L-shaped |
| XJS | 0.441 | L-shaped |
| MDI | 0.627 | L-shaped |
| YJI | 0.025 | shifted |
| MFI | 0.540 | L-shaped |
| ZMF | 0.792 | L-shaped |
| B2I | 0.650 | L-shaped |
| B3I | 0.759 | L-shaped |
| B4I | 0.735 | L-shaped |
| B5I | 0.967 | L-shaped |
| Neighbor mainland populations in TIL | | |
| NOR | 0.873 | L-shaped |
| EAS | 0.952 | L-shaped |
| SOU | 0.785 | L-shaped |

^†^ The TIL-M represents neighbor mainland populations in the TIL region, which was regarded as an mountain population

L-shaped means that population is under mutation-drift equilibrium, indicating no recent bottleneck; shifted means that population is departure from mutation-drift equilibrium, indicating recent bottleneck

**Table S6** Hierarchy analysis of molecular variance for *A. chinensis* populations

| Scales | Source of variance | df | Percentage of variance | *F_ST_* |
| --- | --- | --- | --- | --- |
| Fine scale | Between TIL and neighbor mainland | 1 | 1% | 0.015^**^ |
|  | Among populations | 12 | 5% | 0.065^**^ |
|  | Within populations | 360 | 94% |  |
| Broad scale | Between ZSA and mountains | 1 | 3% | 0.023^**^ |
|  | Among populations | 3 | 7% | 0.095^**^ |
|  | Within populations | 145 | 90% |  |

**Table S7** Parameter statistics for demographic scenario

| Parameter | Mean | Median | Mode | Quantile 2.50% | Quantile 25.00% | Quantile 75.00% | Quantile 97.50% |
| --- | --- | --- | --- | --- | --- | --- | --- |
| TIL island populations (Pop 1) | 9040 | 9180 | 9370 | 7270 | 8740 | 9520 | 9920 |
| TIL-M^†^ populations (Pop 2) | 7420 | 7500 | 7960 | 4450 | 6580 | 8320 | 9700 |
| Mountain populations (Pop 3) | 9990 | 9990 | 9990 | 9980 | 9990 | 10000 | 10000 |
| ZSA population (Pop 4) | 1470 | 1170 | 648 | 459 | 825 | 1700 | 4380 |
| t1 | 62 | 46 | 43 | 19 | 34 | 64 | 213 |
| t2 | 2450 | 1920 | 1290 | 546 | 1240 | 3050 | 7480 |
| t3 | 8880 | 9020 | 9210 | 7040 | 8530 | 9400 | 9900 |

^†^ The TIL-M represents neighbor mainland populations in the TIL region, which was regarded as an mountain population

Pop 1 and Pop 2 diverged at t1; Pop 4 diverged at t2; Pop 3 diverged at t3

**Table S8** Correlation analysis between genetic diversity, population size and island features

|  | *Ae* | *He* | PS | Area | Perimeter | SI | DTI | DTL |
| --- | --- | --- | --- | --- | --- | --- | --- | --- |
| *Ae* |  | 0.973^**^ | 0.405 | 0.387 | 0.420 | 0.124 | 0.241 | -0.038 |
| *He* |  |  | 0.396 | 0.342 | 0.370 | 0.172 | 0.252 | 0.029 |
| PS |  |  |  | 0.963^**^ | 0.943^**^ | -0.168 | -0.276 | 0.178 |
| Area |  |  |  |  | 0.987^**^ | -0.263 | -0.237 | 0.089 |
| Perimeter |  |  |  |  |  | -0.210 | -0.217 | 0.057 |
| SI |  |  |  |  |  |  | -0.052 | -0.274 |
| DTI |  |  |  |  |  |  |  | -0.416 |
| DTL |  |  |  |  |  |  |  |  |

Abbreviations: *Ae*, number of effective alleles; *He*, expected heterozygosity; PS, population size; SI, $shape index=P/\left[ 2\times\left( \pi\times A \right)^{0.5} \right]$; DTI, distance to the nearest island; DTL, distance to the nearest mainland

**Table S9** Matrix of pairwise *F_ST_* and *Nm* coefficient of *A. chinensis* populations in TIL

| JSI | MGI | XJS | MDI | YJI | MFI | ZMF | B2I | B3I | B4I | B5I | NOR | EAS | SOU |  |
| --- | --- | --- | --- | --- | --- | --- | --- | --- | --- | --- | --- | --- | --- | --- |
|  | 0.017 | 0.067 | 0.048 | 0.053 | 0.026 | 0.052 | 0.045 | 0.074 | 0.050 | 0.073 | 0.015 | 0.059 | 0.091 | JSI |
| 14.569 |  | 0.051 | 0.069 | 0.095 | 0.027 | 0.044 | 0.035 | 0.060 | 0.045 | 0.066 | 0.023 | 0.036 | 0.070 | MGI |
| 3.487 | 4.652 |  | 0.102 | 0.153 | 0.072 | 0.084 | 0.045 | 0.058 | 0.046 | 0.062 | 0.055 | 0.042 | 0.086 | XJS |
| 4.932 | 3.381 | 2.194 |  | 0.052 | 0.069 | 0.114 | 0.069 | 0.087 | 0.068 | 0.087 | 0.024 | 0.088 | 0.131 | MDI |
| 4.465 | 2.376 | 1.383 | 4.603 |  | 0.076 | 0.119 | 0.101 | 0.125 | 0.098 | 0.124 | 0.052 | 0.124 | 0.163 | YJI |
| 9.185 | 9.164 | 3.208 | 3.394 | 3.019 |  | 0.044 | 0.027 | 0.059 | 0.026 | 0.065 | 0.033 | 0.077 | 0.099 | MFI |
| 4.533 | 5.438 | 2.710 | 1.948 | 1.851 | 5.422 |  | 0.062 | 0.100 | 0.069 | 0.108 | 0.051 | 0.072 | 0.077 | ZMF |
| 5.298 | 6.902 | 5.324 | 3.380 | 2.232 | 8.915 | 3.804 |  | 0.003^†^ | 0.000^†^ | 0.016 | 0.028 | 0.047 | 0.079 | B2I |
| 3.148 | 3.916 | 4.045 | 2.620 | 1.746 | 3.953 | 2.249 | 82.245 |  | 0.007^†^ | 0.011^†^ | 0.038 | 0.043 | 0.075 | B3I |
| 4.764 | 5.264 | 5.171 | 3.446 | 2.312 | 9.358 | 3.380 | N/A^††^ | 34.376 |  | 0.009^†^ | 0.031 | 0.052 | 0.091 | B4I |
| 3.170 | 3.526 | 3.770 | 2.622 | 1.767 | 3.572 | 2.068 | 15.069 | 22.618 | 28.819 |  | 0.045 | 0.056 | 0.091 | B5I |
| 16.334 | 10.791 | 4.315 | 10.020 | 4.577 | 7.220 | 4.657 | 8.628 | 6.257 | 7.860 | 5.281 |  | 0.021 | 0.050 | NOR |
| 4.016 | 6.628 | 5.739 | 2.592 | 1.772 | 2.991 | 3.244 | 5.063 | 5.577 | 4.544 | 4.202 | 11.521 |  | 0.020 | EAS |
| 2.497 | 3.325 | 2.649 | 1.662 | 1.285 | 2.269 | 2.997 | 2.924 | 3.073 | 2.494 | 2.499 | 4.797 | 12.236 |  | SOU |

^†^ No significant differentiation was found between the pair of populations

^††^ N/A means the *Nm* value between B2I and B4I populations cannot be calculated because the *F_ST_* value is zero

Top-right matrix refers to pairwise genetic differentiation coefficient. Lower-left matrix refers to pairwise gene flow coefficient

| 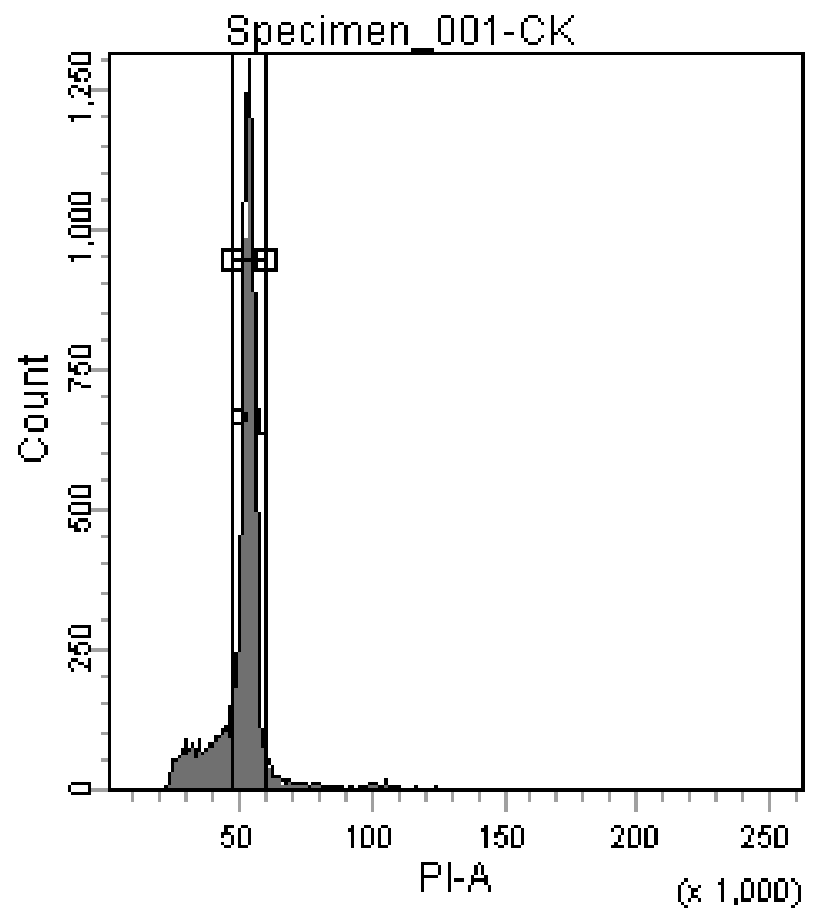 | 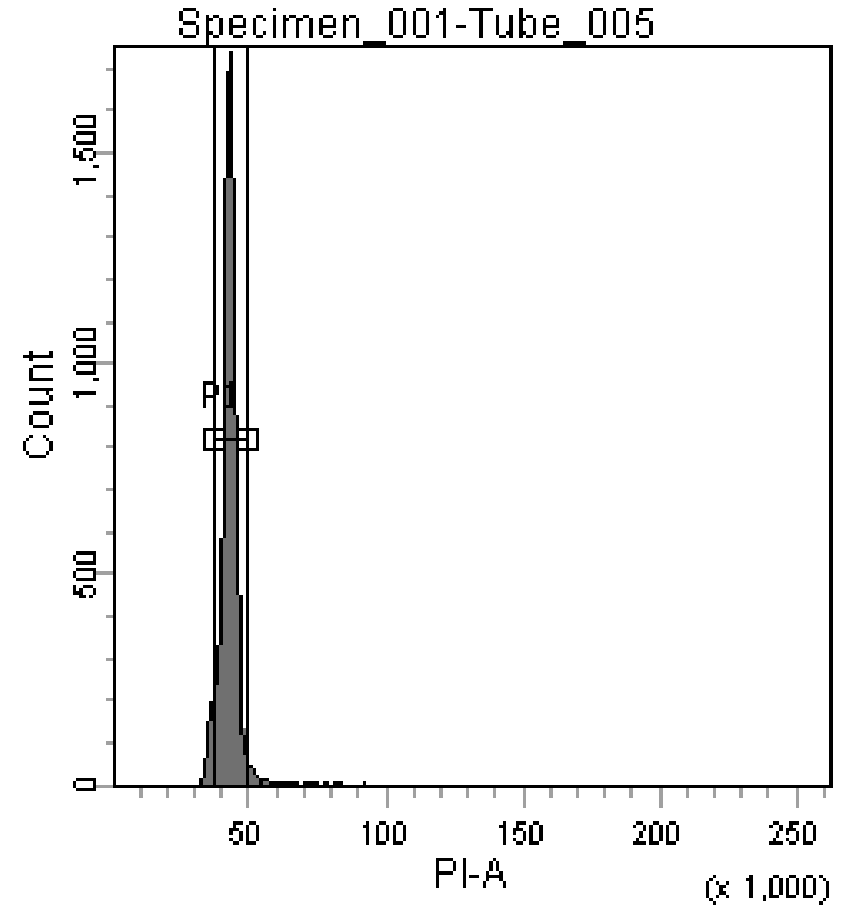 |
| --- | --- |
| (A) Control Group | (B) Diploid in XHZ population |
| 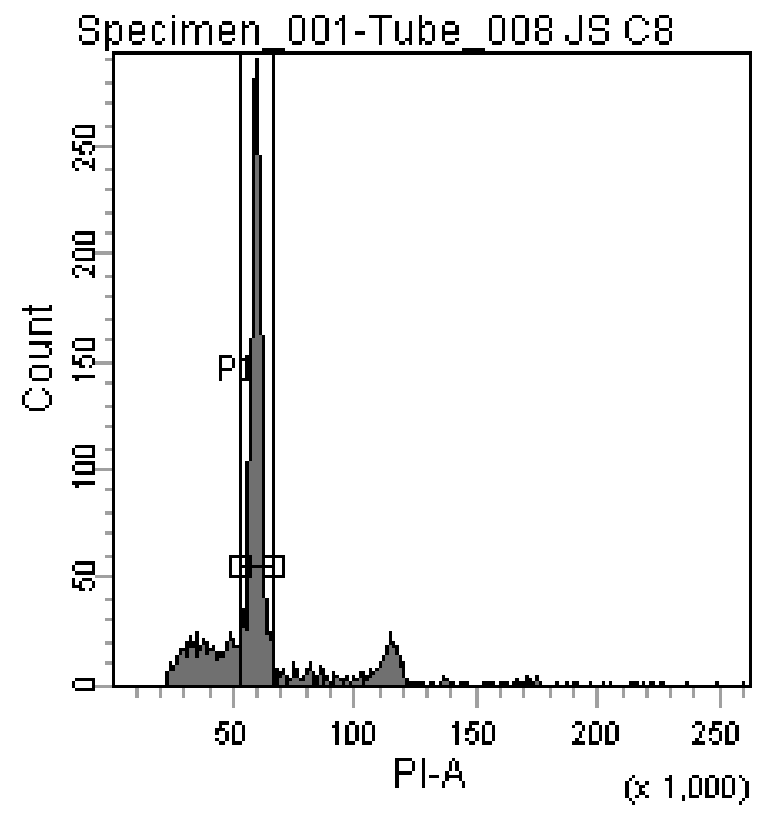 | 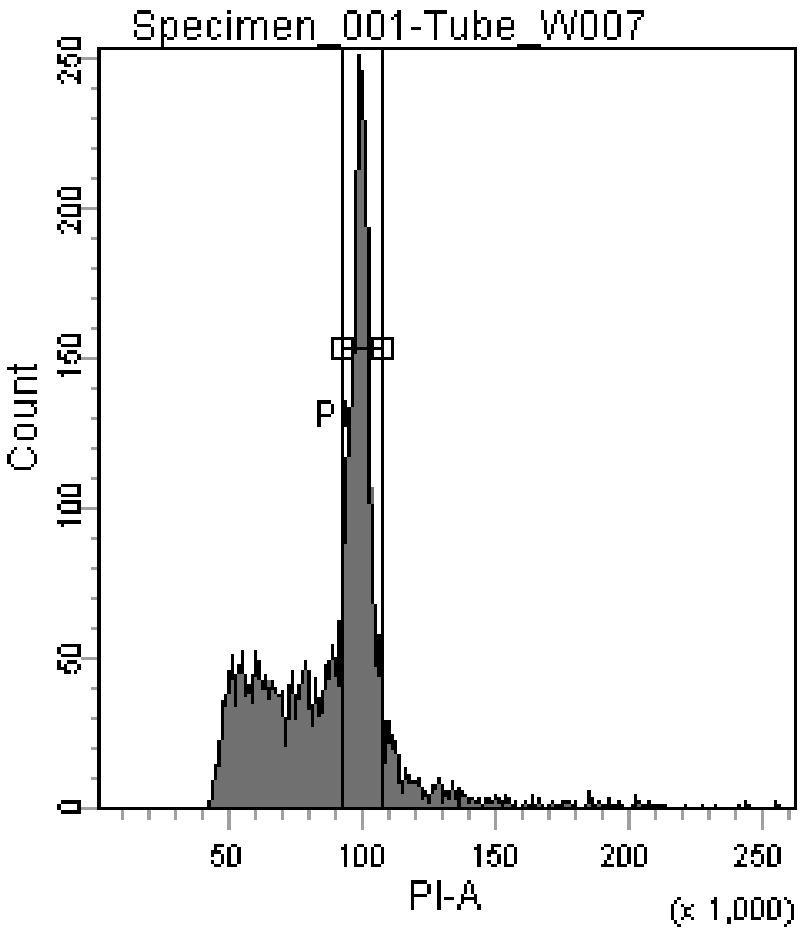 |
| (C) Diploid in JSI population | (D) Tetraploid in ZSA population |

**Fig. S1** Partial results of chromosome ploidy detection of *A. chinensis*. The PI-A values represent the size of genome (PI-A=50, diploid; PI-A=100, tetraploid; PI-A=150, hexaploid; PI-A= 200, octaploid)


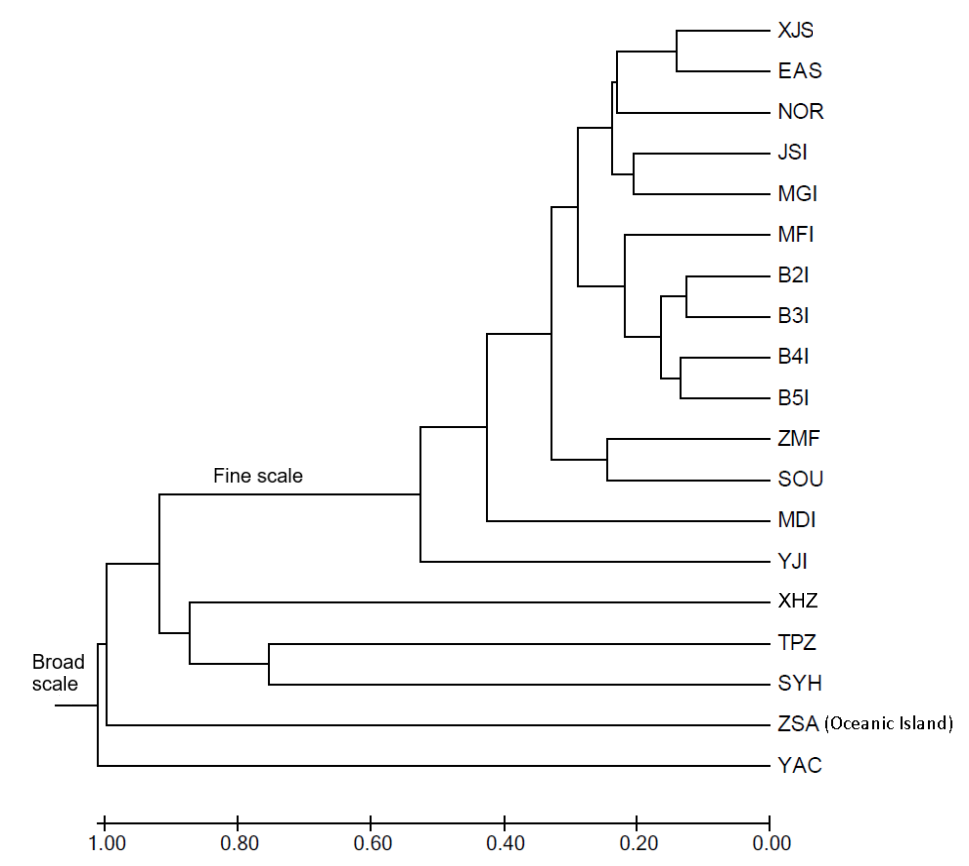


**Fig. S2** Cluster analysis by unweighted pair-group method with arithmetic means based on Nei’ genetic distance of *A. chinensis* populations

**
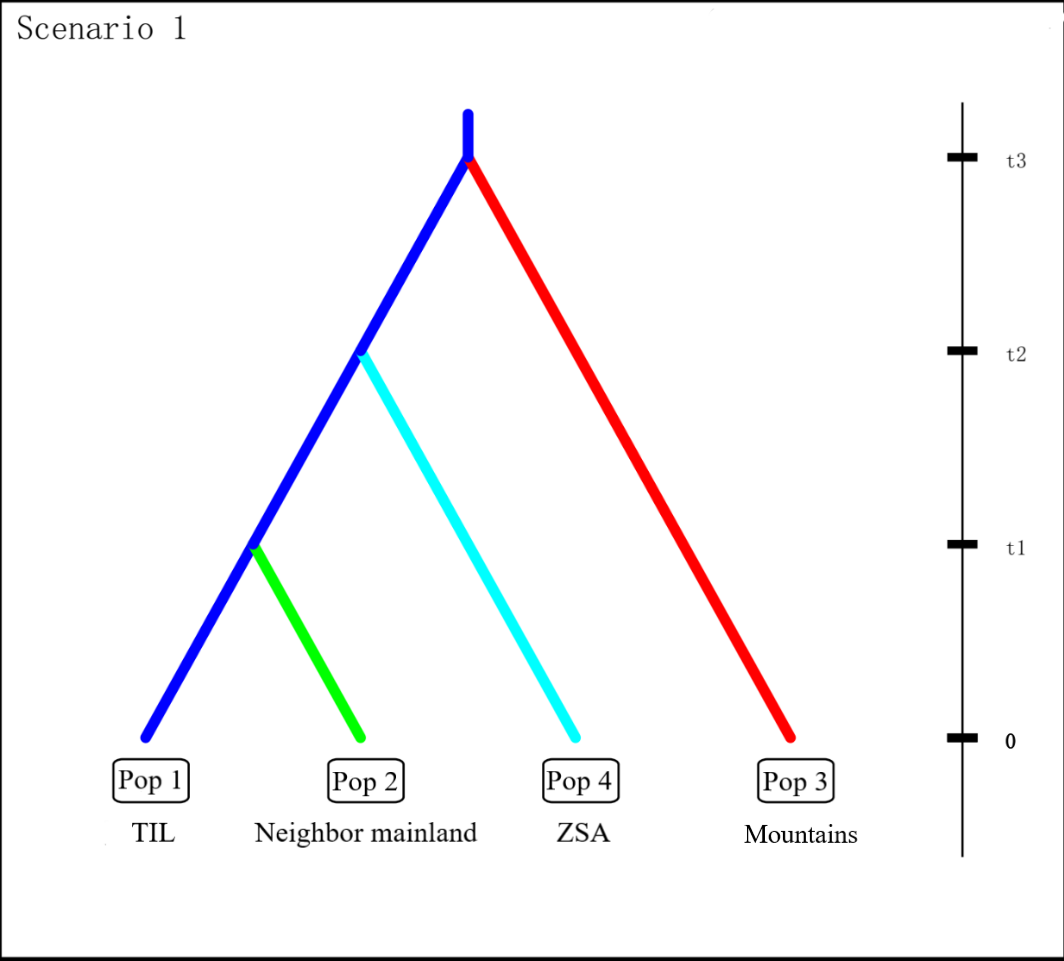
**

**Fig. S3** Demographic scenario tested in DIYABC simulations. Effective population size and divergence time (t1-3) for the four genetic clusters, i.e. TIL populations (Pop 1, dark blue line), ZSA population (Pop 4, light blue line), neighbor mainland (Pop 2, green line), and mountain populations (Pop 3, red line). Scenario 1 was a demographic scenario that was set to infer population history. Pop 1 refers to eleven TIL island populations; Pop 2 refers to three neighbor mainland populations around TIL (NOR, SOU, EAS); Pop 3 refers to the mountain populations (TPZ, XHZ, SYH, YAC); Pop 4 refers to the oceanic ZSA island population.


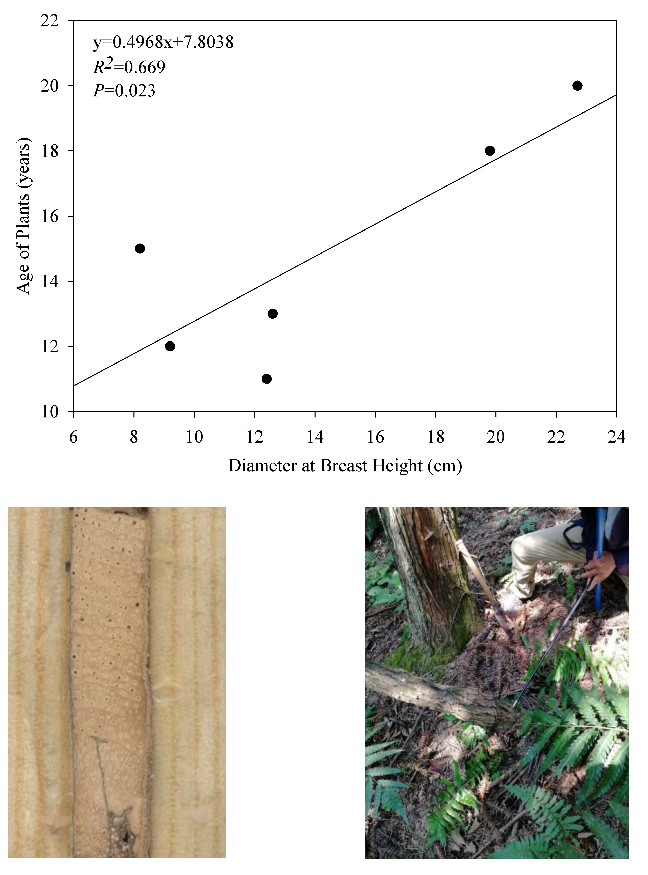


**Fig. S4** Linear regression analysis between diameter at breast height and age of plants
